# Supplementary material for: Antimicrobial susceptibility patterns of respiratory Gram-negative bacterial isolates from COVID-19 patients in Switzerland
Source: Ann Clin Microbiol Antimicrob. 2021 Sep 7;20:64. doi: 10.1186/s12941-021-00468-1 (PMC8422836; doi:10.1186/s12941-021-00468-1)
Supplement: Supplementary file 1 — Additional file 1. Additional figures S1–S4 and Tables S1–S4. [file 12941_2021_468_MOESM1_ESM.pdf]

Supplementary Information

Antimicrobial susceptibility patterns of respiratory Gram-negative bacterial isolates from COVID-19 patients in Switzerland

Marina Gysin[a], Claudio Tirso Acevedo[b]*, Klara Haldimann[a]*, Elias Bodendoerfer[a], Frank Imkamp[a], Karl Bulut[a], Philipp Karl Buehler[c], Silvio Daniel Brugger[b], Katja Becker[a], Sven N. Hobbie[a]***

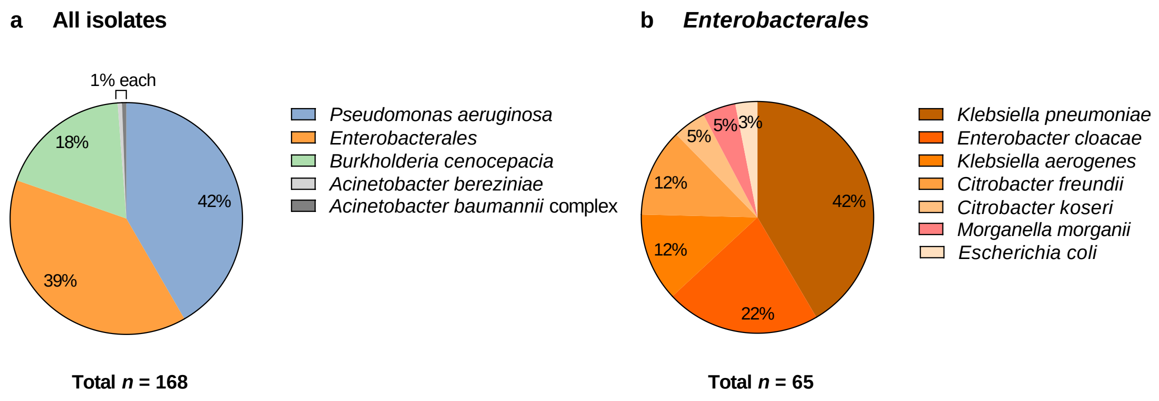

Figure S1. MicrobiotaCOVID cohort species distribution of all identified Gram-negative isolates (a) and within *Enterobacterales* (b).

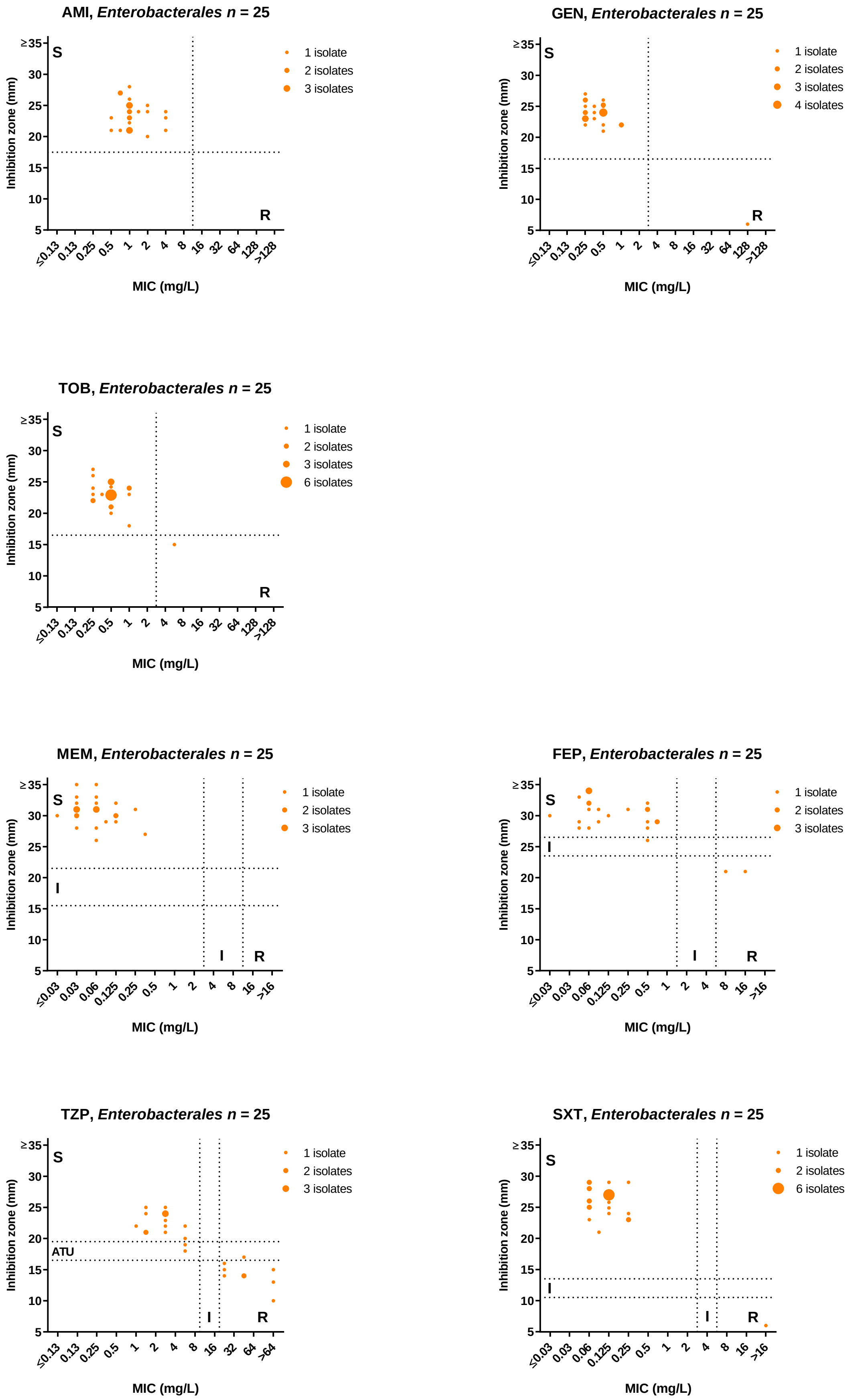

Figure S2. Correlation plots of EUCAST disc diffusion and CLSI broth microdilution testing for *Enterobacterales*. Amikacin (AMI), gentamicin (GEN), tobramycin (TOB), meropenem (MEM), cefepime (FEP), piperacillin/tazobactam (TZP) and trimethoprim/sulfamethoxazole (SXT).

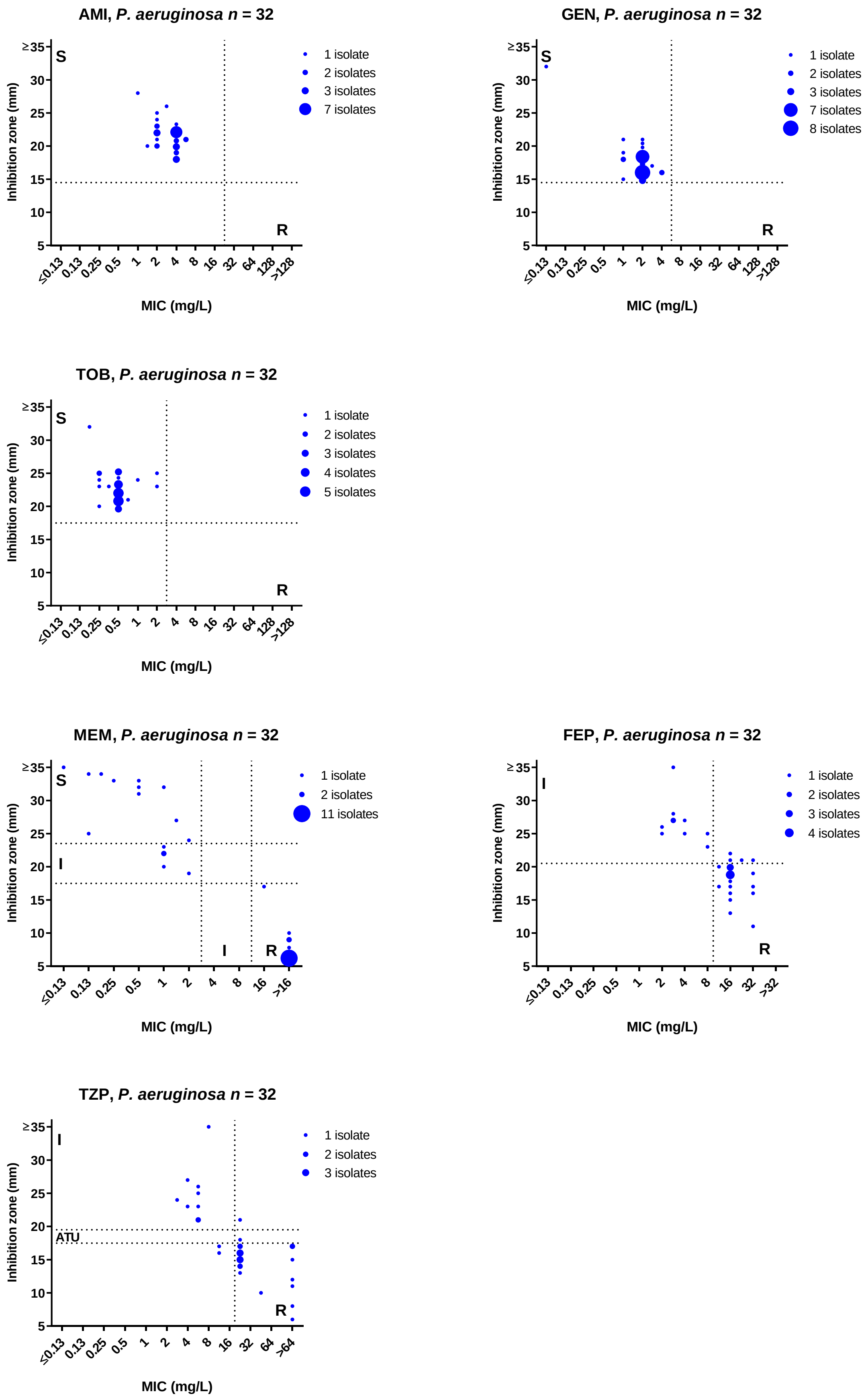

Figure S3. Correlation plots of EUCAST disc diffusion and CLSI broth microdilution testing for *P. aeruginosa*. Amikacin (AMI), gentamicin (GEN), tobramycin (TOB), meropenem (MEM), cefepime (FEP) and piperacillin/tazobactam (TZP).

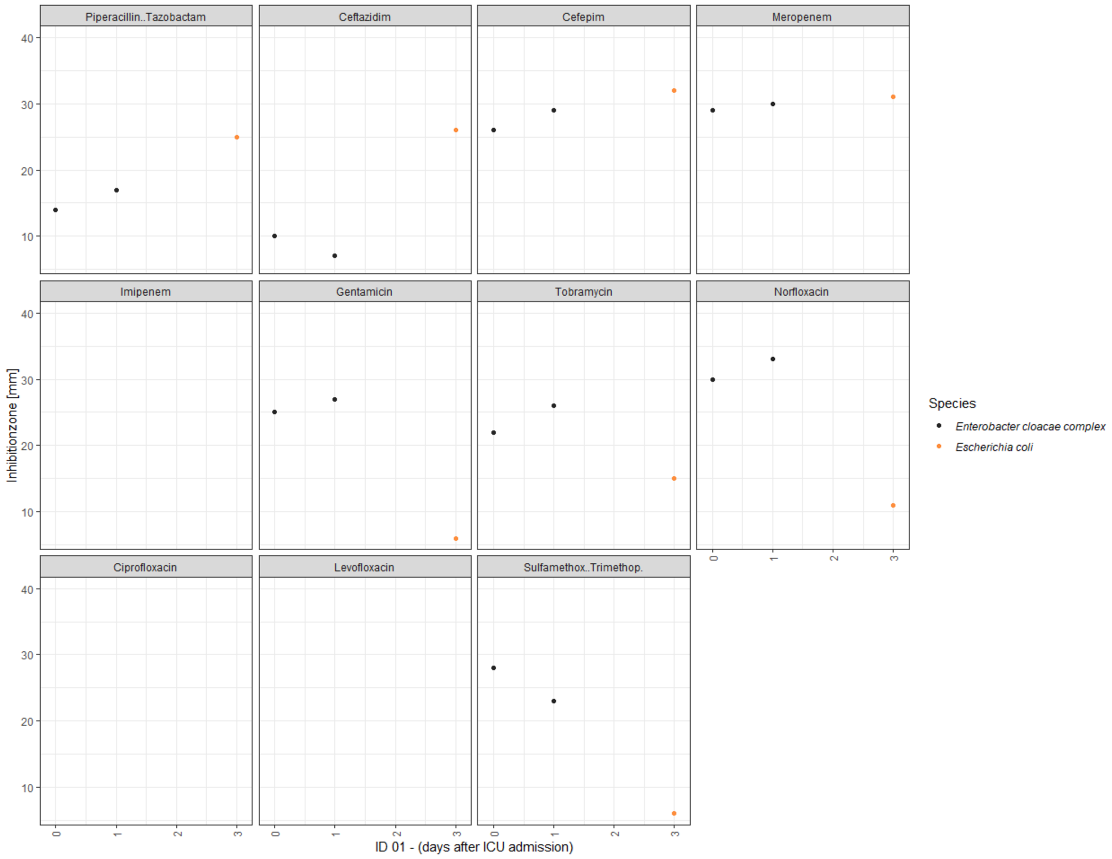

Piperacillin..Tazobactam
Ceftazidim
Cefepim
Meropenem
Imipenem
Gentamicin
Tobramycin
Norfloxacin
Ciprofloxacin
Levofloxacin
Sulfamethox..Trimethop.
Inhibitionzone [mm]
ID 01 - (days after ICU admission)
Species
Enterobacter cloacae complex
Escherichia coli

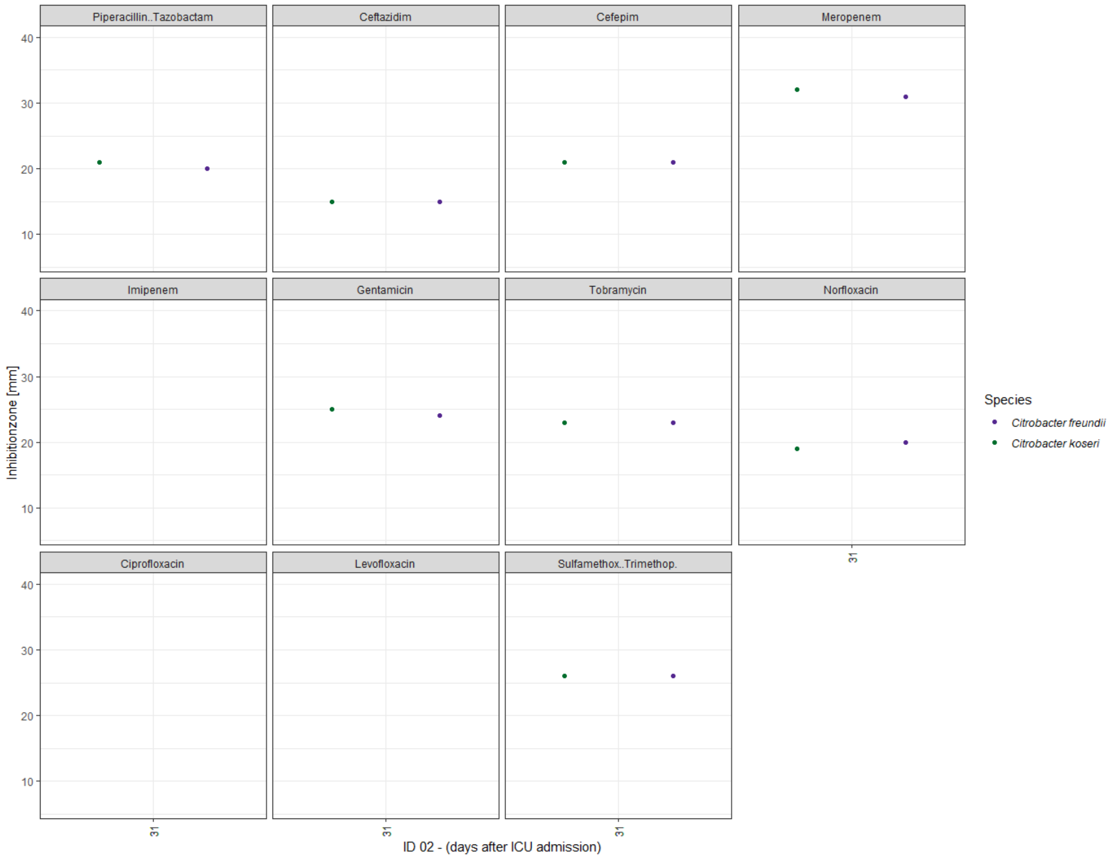

Piperacillin..Tazobactam
Ceftazidim
Cefepim
Meropenem
Imipenem
Gentamicin
Tobramycin
Norfloxacin
Ciprofloxacin
Levofloxacin
Sulfamethox..Trimethop.
Inhibitionzone [mm]
ID 02 - (days after ICU admission)
Species
Citrobacter freundii
Citrobacter koseri

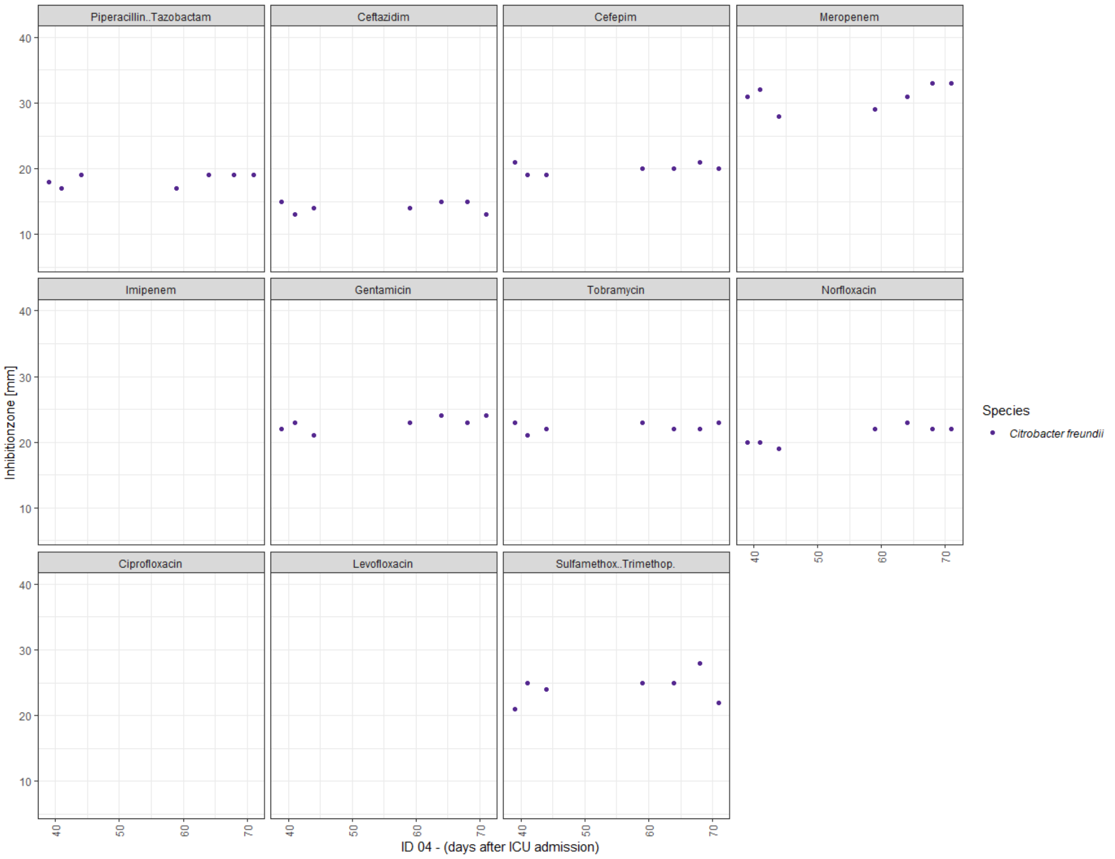

Piperacillin..Tazobactam
Ceftazidim
Cefepim
Meropenem
Imipenem
Gentamicin
Tobramycin
Norfloxacin
Ciprofloxacin
Levofloxacin
Sulfamethox..Trimethop.
Inhibitionzone [mm]
ID 04 - (days after ICU admission)
Species
Citrobacter freundii

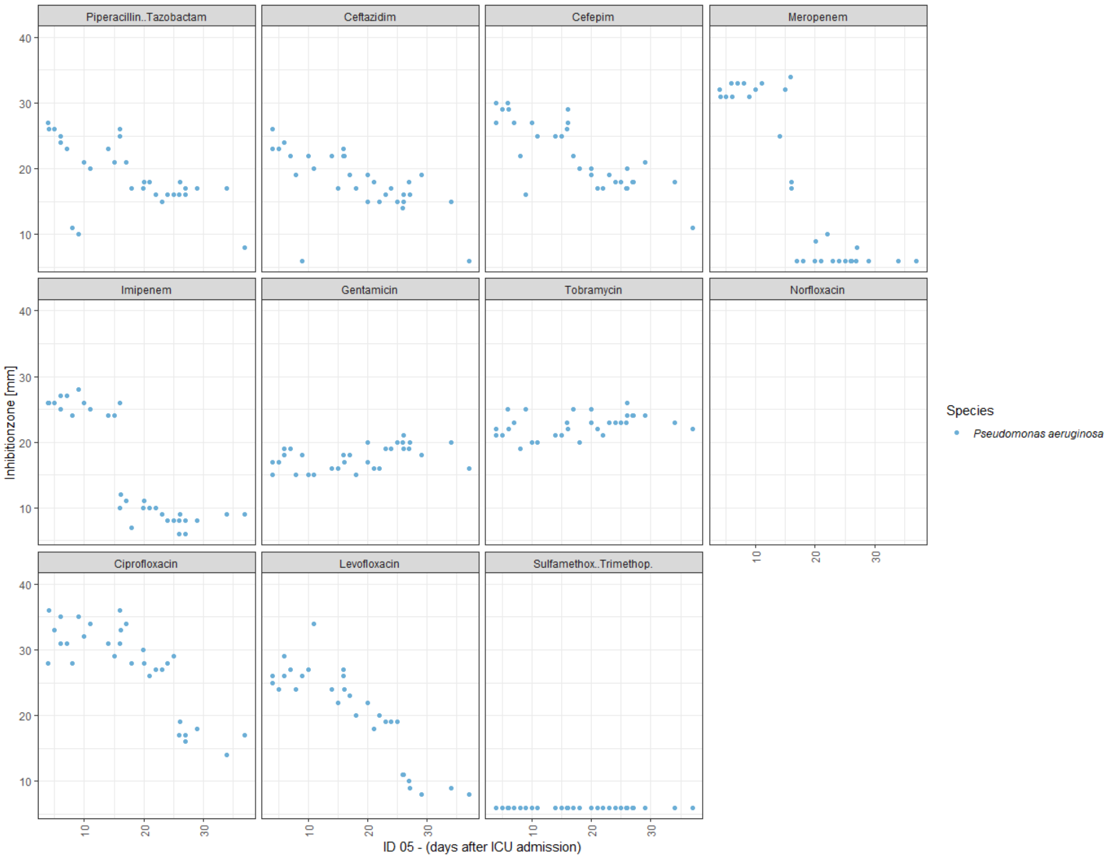

Piperacillin..Tazobactam
Ceftazidim
Cefepim
Meropenem
Imipenem
Gentamicin
Tobramycin
Norfloxacin
Ciprofloxacin
Levofloxacin
Sulfamethox..Trimethop.
Inhibitionzone [mm]
ID 05 - (days after ICU admission)
Species
Pseudomonas aeruginosa

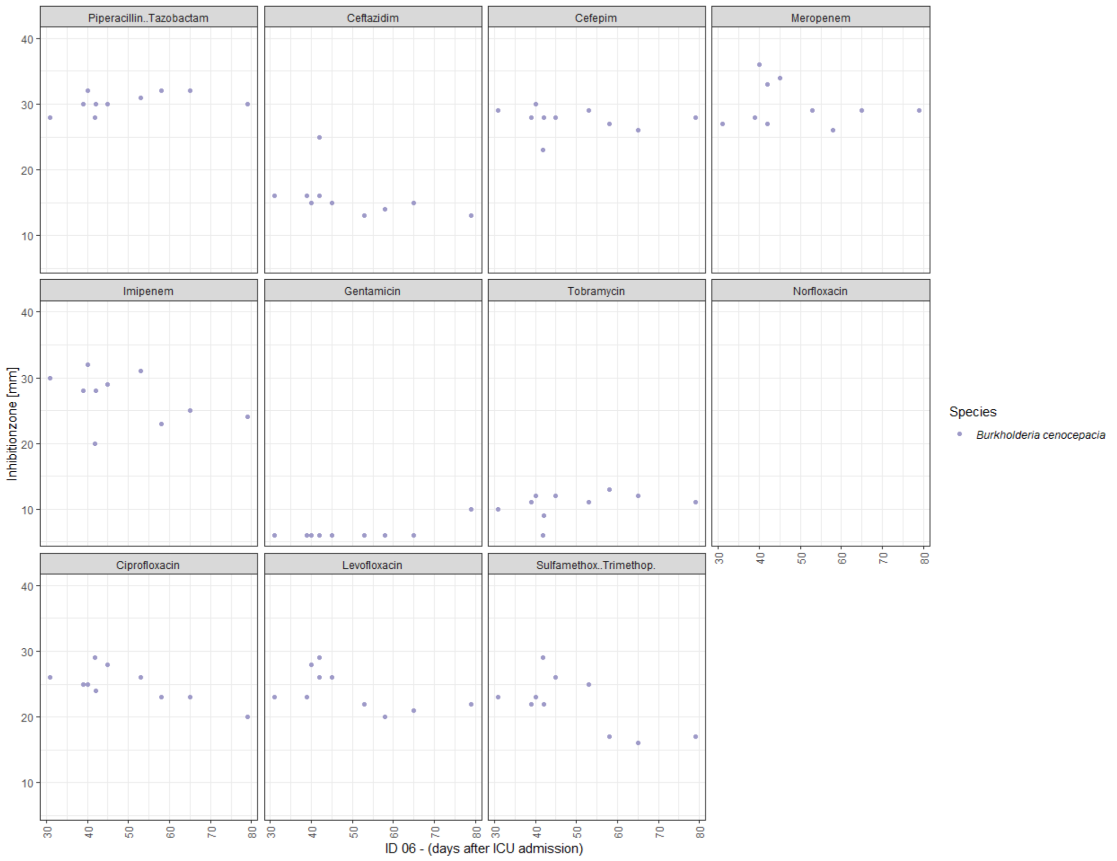
Piperacillin..Tazobactam
Ceftazidim
Cefepim
Meropenem
Imipenem
Gentamicin
Tobramycin
Norfloxacin
Ciprofloxacin
Levofloxacin
Sulfamethox..Trimethop.
Inhibitionzone [mm]
ID 06 - (days after ICU admission)
Species
Burkholderia cenocepacia

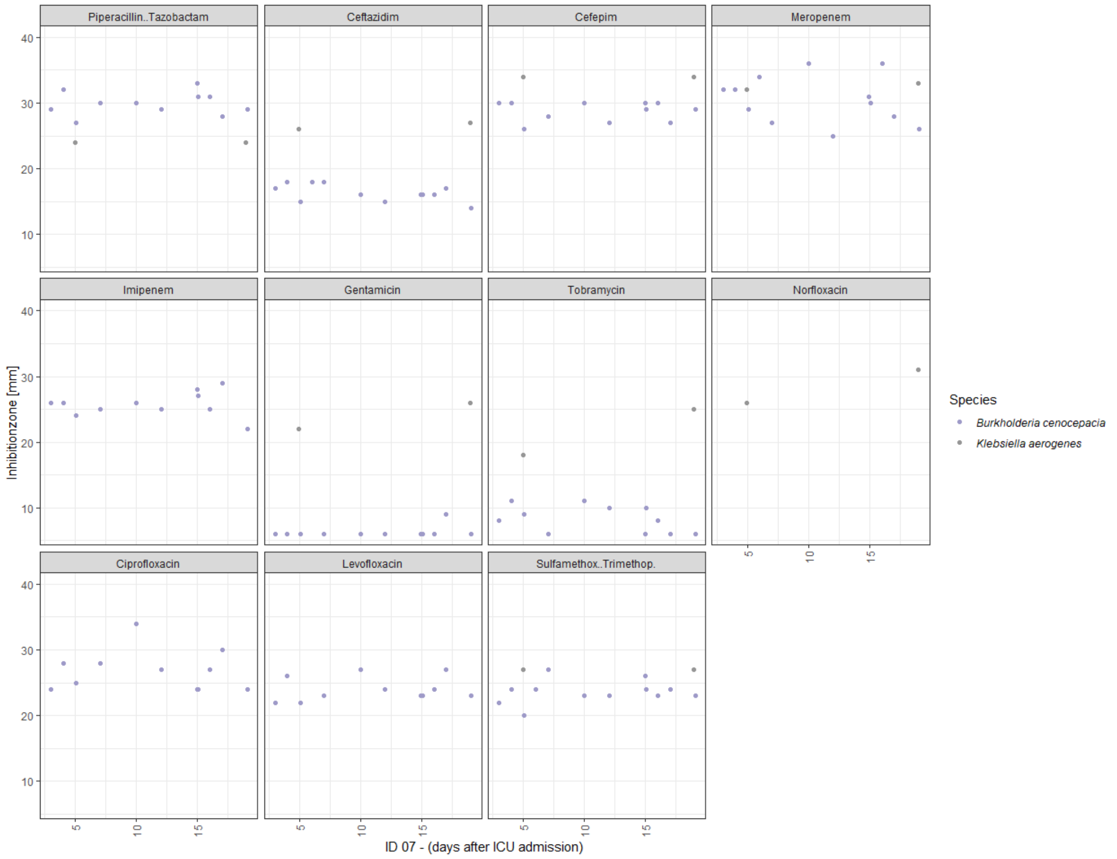
Piperacillin..Tazobactam
Ceftazidim
Cefepim
Meropenem
Imipenem
Gentamicin
Tobramycin
Norfloxacin
Ciprofloxacin
Levofloxacin
Sulfamethox..Trimethop.
Inhibitionzone [mm]
ID 07 - (days after ICU admission)
Species
Burkholderia cenocepacia
Klebsiella aerogenes

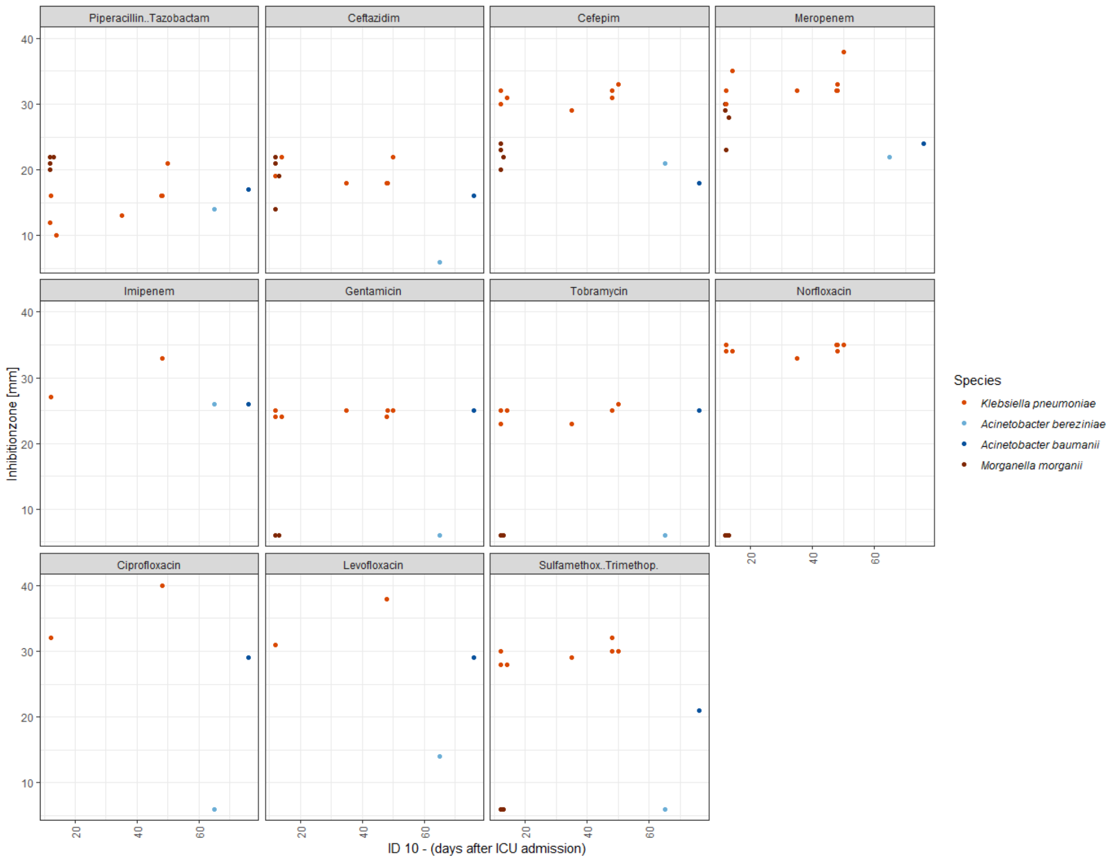
Piperacillin..Tazobactam
Ceftazidim
Cefepim
Meropenem
Imipenem
Gentamicin
Tobramycin
Norfloxacin
Ciprofloxacin
Levofloxacin
Sulfamethox..Trimethop.
Inhibitionzone [mm]
ID 10 - (days after ICU admission)
Species
Klebsiella pneumoniae
Acinetobacter bereziniae
Acinetobacter baumanii
Morganella morganii

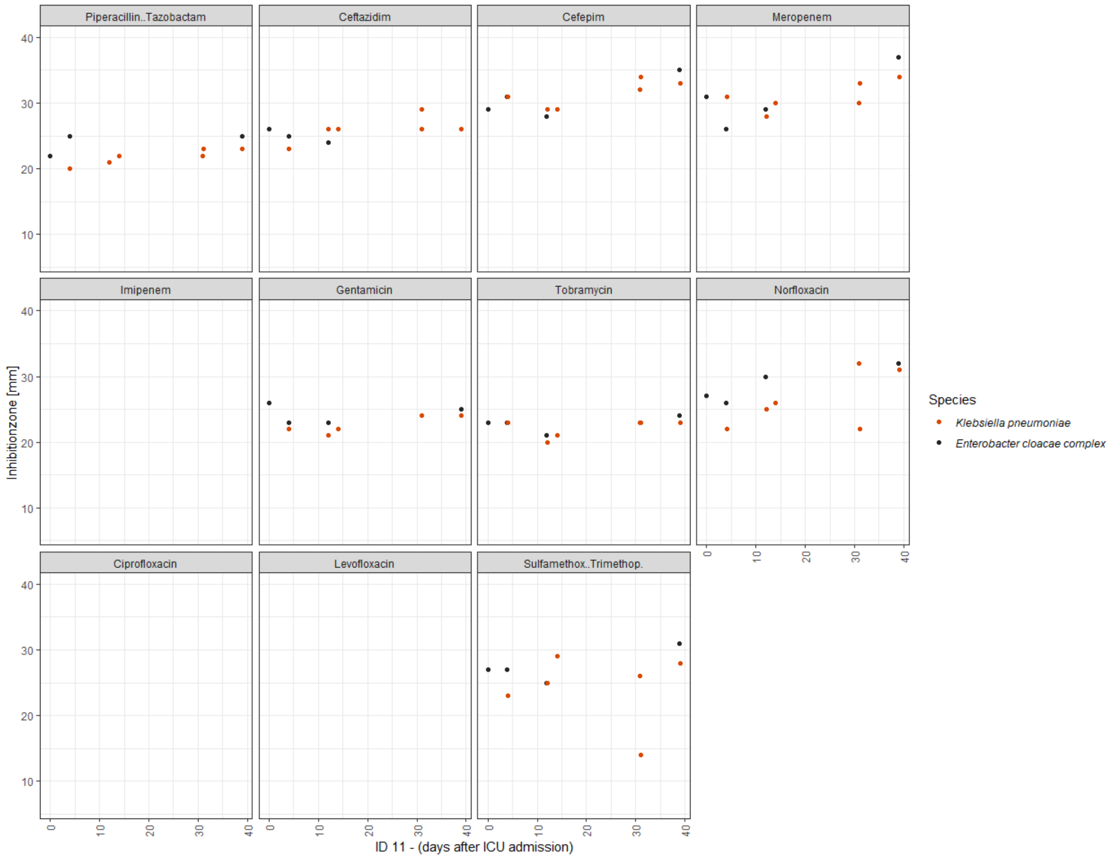
Piperacillin..Tazobactam
Ceftazidim
Cefepim
Meropenem
Imipenem
Gentamicin
Tobramycin
Norfloxacin
Ciprofloxacin
Levofloxacin
Sulfamethox..Trimethop.
Inhibitionzone [mm]
ID 11 - (days after ICU admission)
Species
Klebsiella pneumoniae
Enterobacter cloacae complex

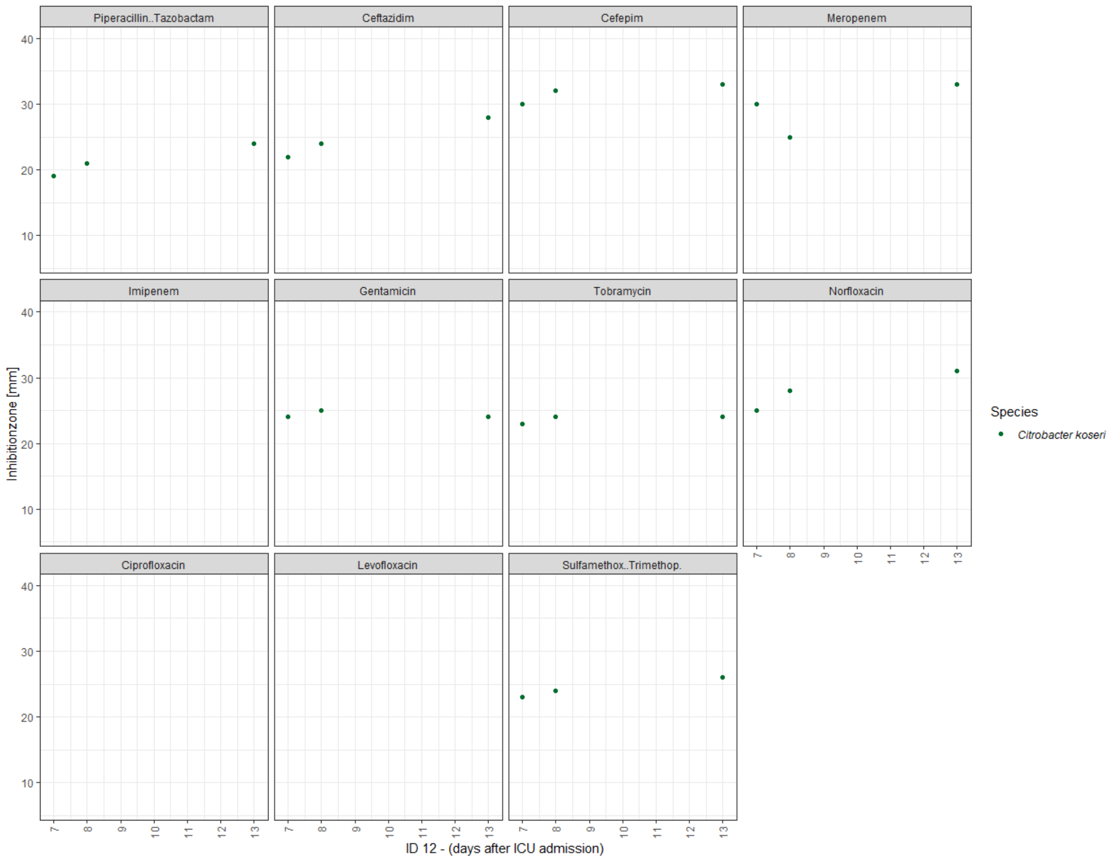
Piperacillin..Tazobactam
Ceftazidim
Cefepim
Meropenem
Imipenem
Gentamicin
Tobramycin
Norfloxacin
Ciprofloxacin
Levofloxacin
Sulfamethox..Trimethop.
Inhibitionzone [mm]
ID 12 - (days after ICU admission)
Species
Citrobacter koseri

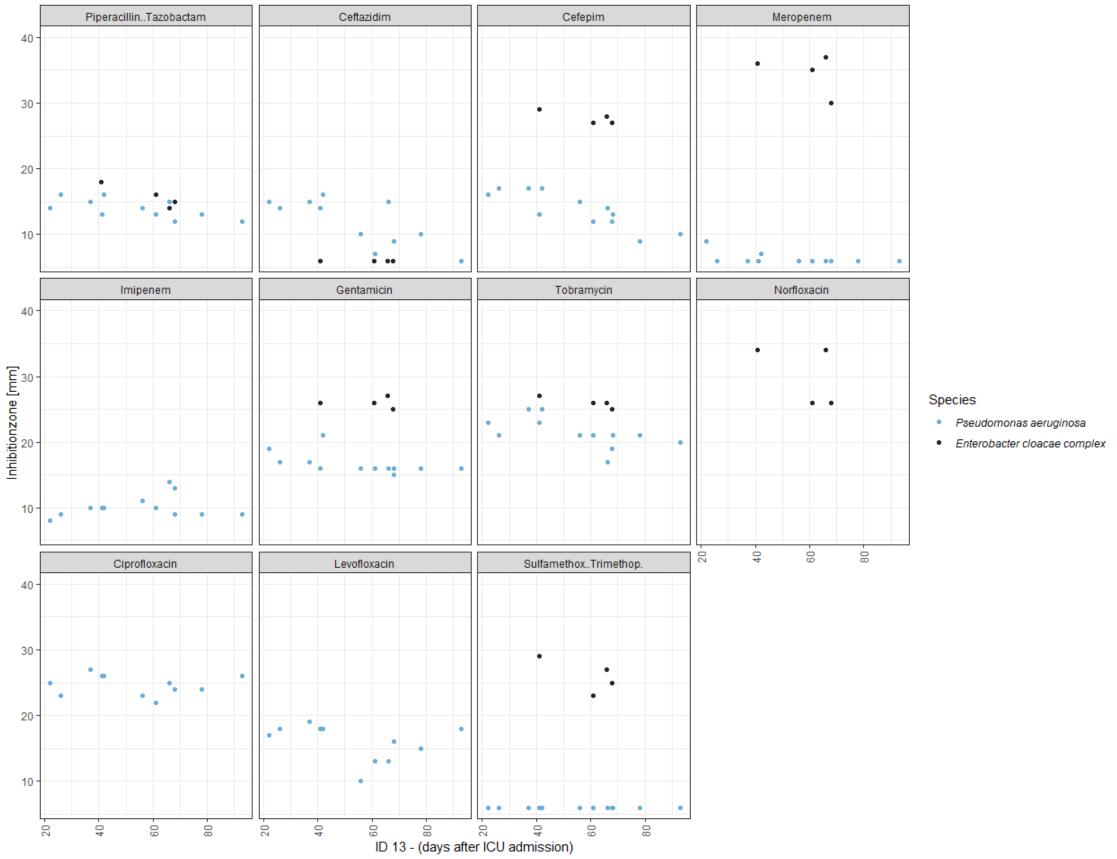
Piperacillin..Tazobactam
Ceftazidim
Cefepim
Meropenem
Imipenem
Gentamicin
Tobramycin
Norfloxacin
Ciprofloxacin
Levofloxacin
Sulfamethox..Trimethop.
Inhibitionzone [mm]
ID 13 - (days after ICU admission)
Species
Pseudomonas aeruginosa
Enterobacter cloacae complex

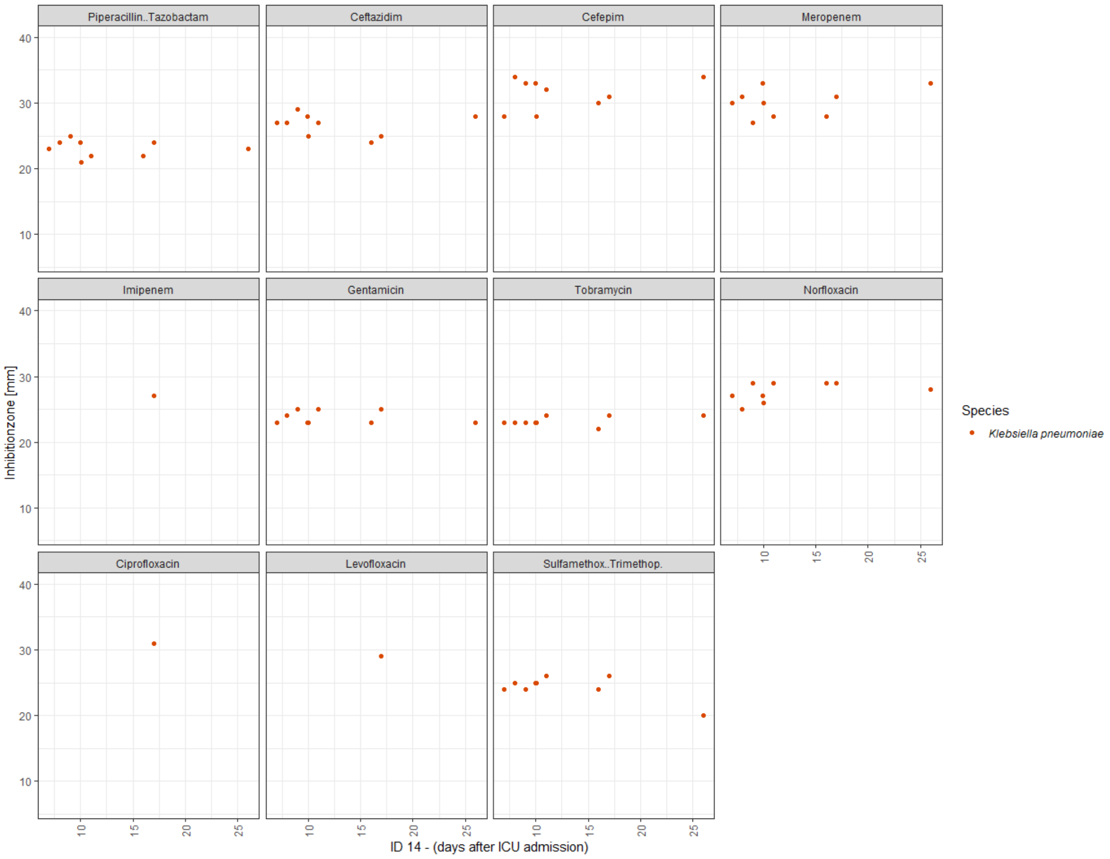
Piperacillin..Tazobactam
Ceftazidim
Cefepim
Meropenem
Imipenem
Gentamicin
Tobramycin
Norfloxacin
Ciprofloxacin
Levofloxacin
Sulfamethox..Trimethop.
Inhibitionzone [mm]
ID 14 - (days after ICU admission)
Species
Klebsiella pneumoniae

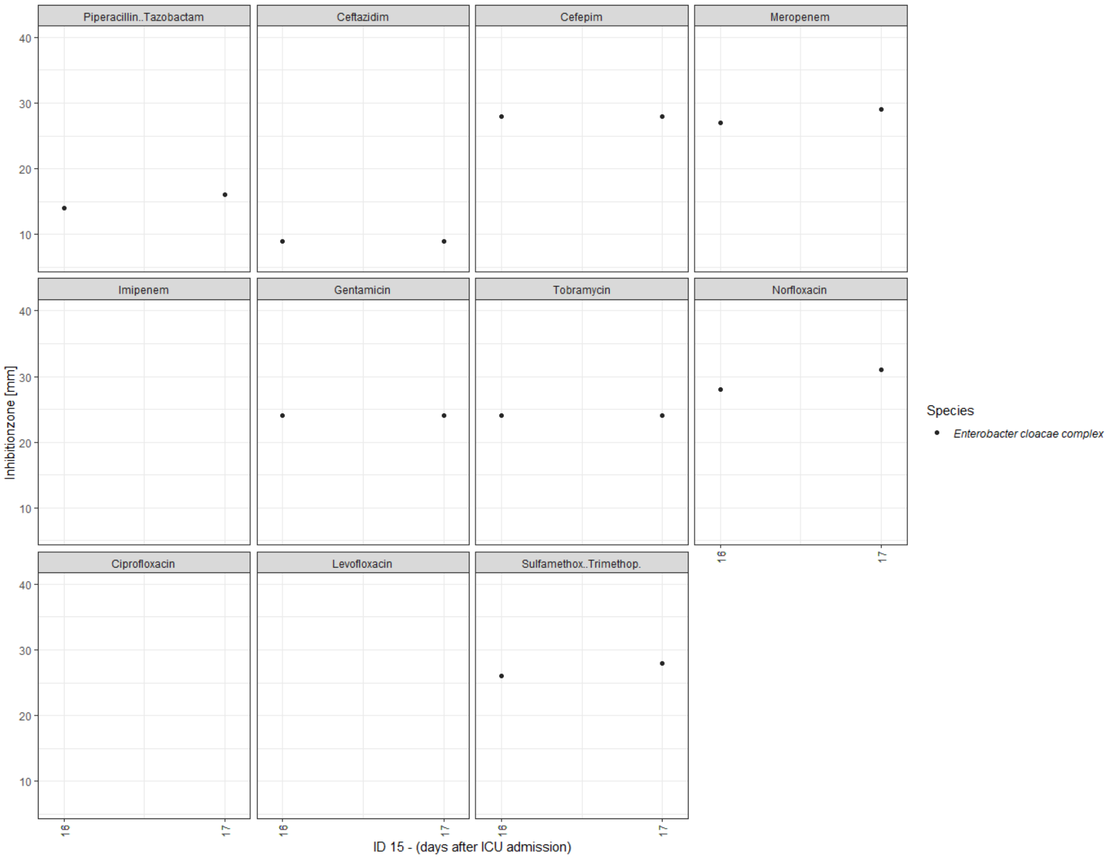
Piperacillin..Tazobactam
Ceftazidim
Cefepim
Meropenem
Imipenem
Gentamicin
Tobramycin
Norfloxacin
Ciprofloxacin
Levofloxacin
Sulfamethox..Trimethop.
Inhibitionzone [mm]
ID 15 - (days after ICU admission)
Species
Enterobacter cloacae complex

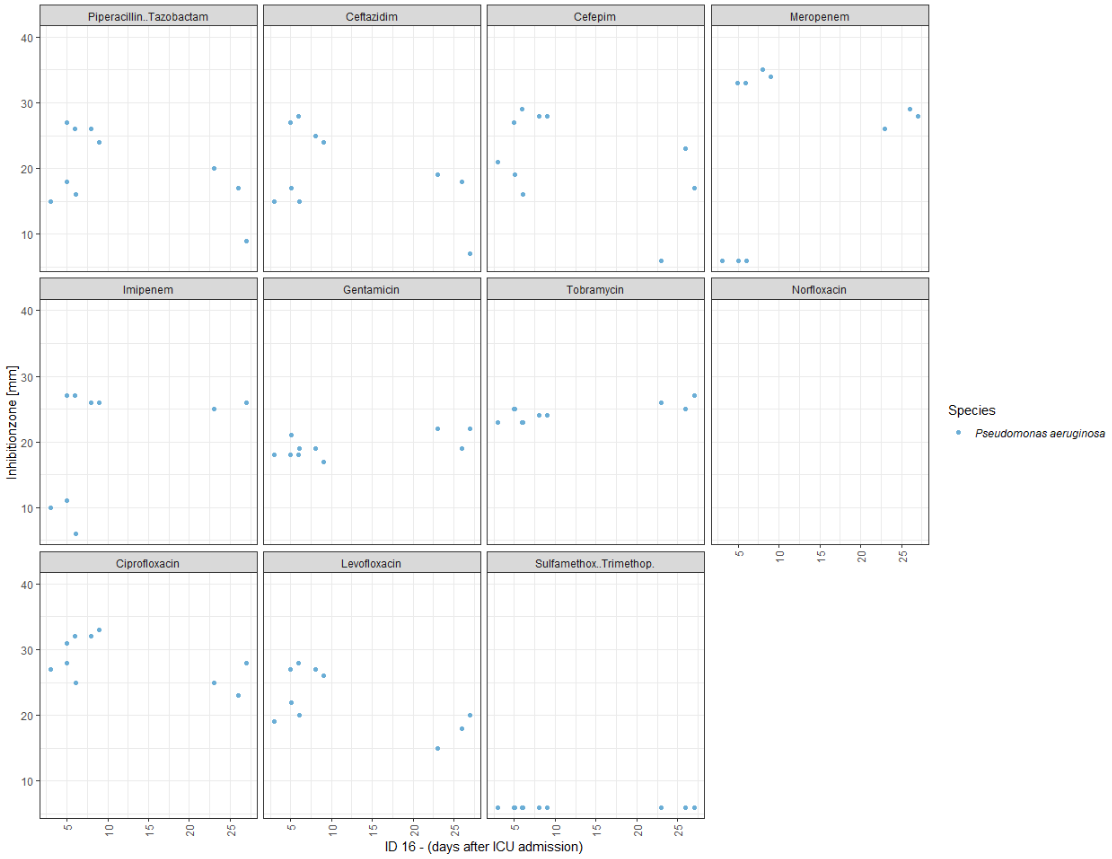
Piperacillin..Tazobactam
Ceftazidim
Cefepim
Meropenem
Imipenem
Gentamicin
Tobramycin
Norfloxacin
Ciprofloxacin
Levofloxacin
Sulfamethox..Trimethop.
Inhibitionzone [mm]
Species
Pseudomonas aeruginosa
ID 16 - (days after ICU admission)

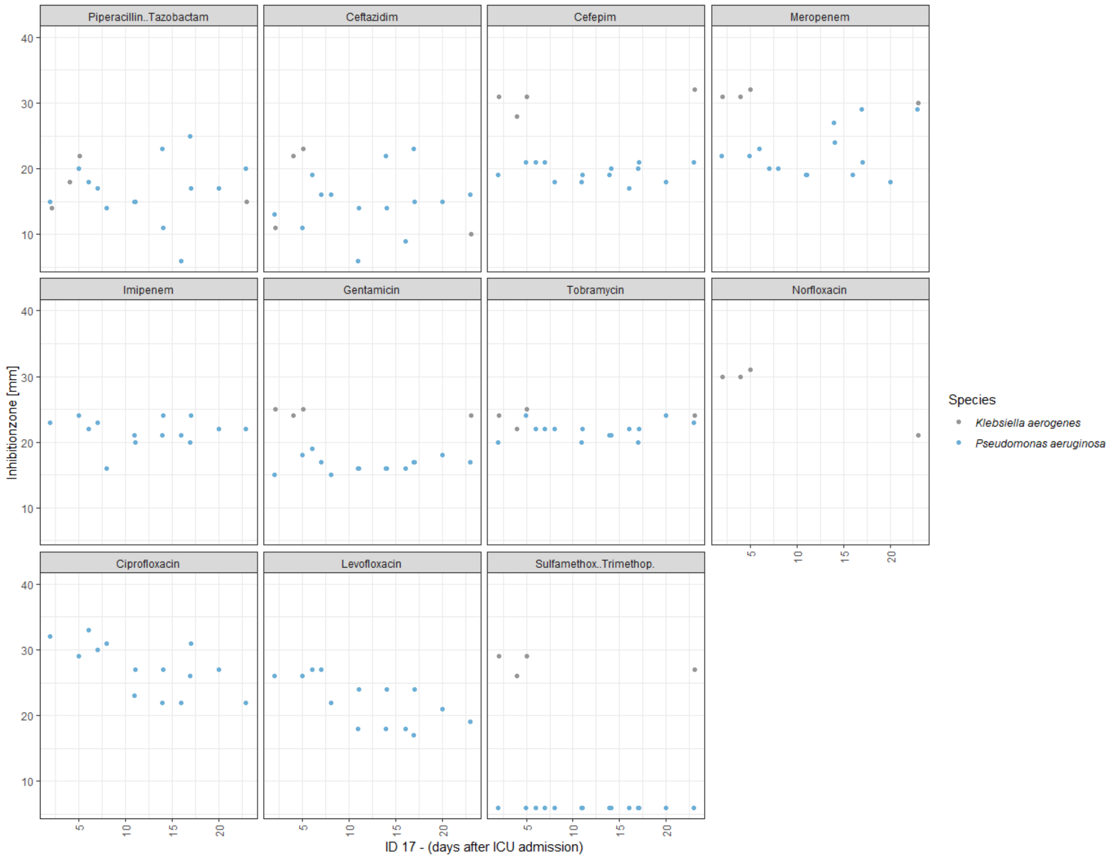
Piperacillin..Tazobactam
Ceftazidim
Cefepim
Meropenem
Imipenem
Gentamicin
Tobramycin
Norfloxacin
Ciprofloxacin
Levofloxacin
Sulfamethox..Trimethop.
Inhibitionzone [mm]
Species
Klebsiella aerogenes
Pseudomonas aeruginosa
ID 17 - (days after ICU admission)

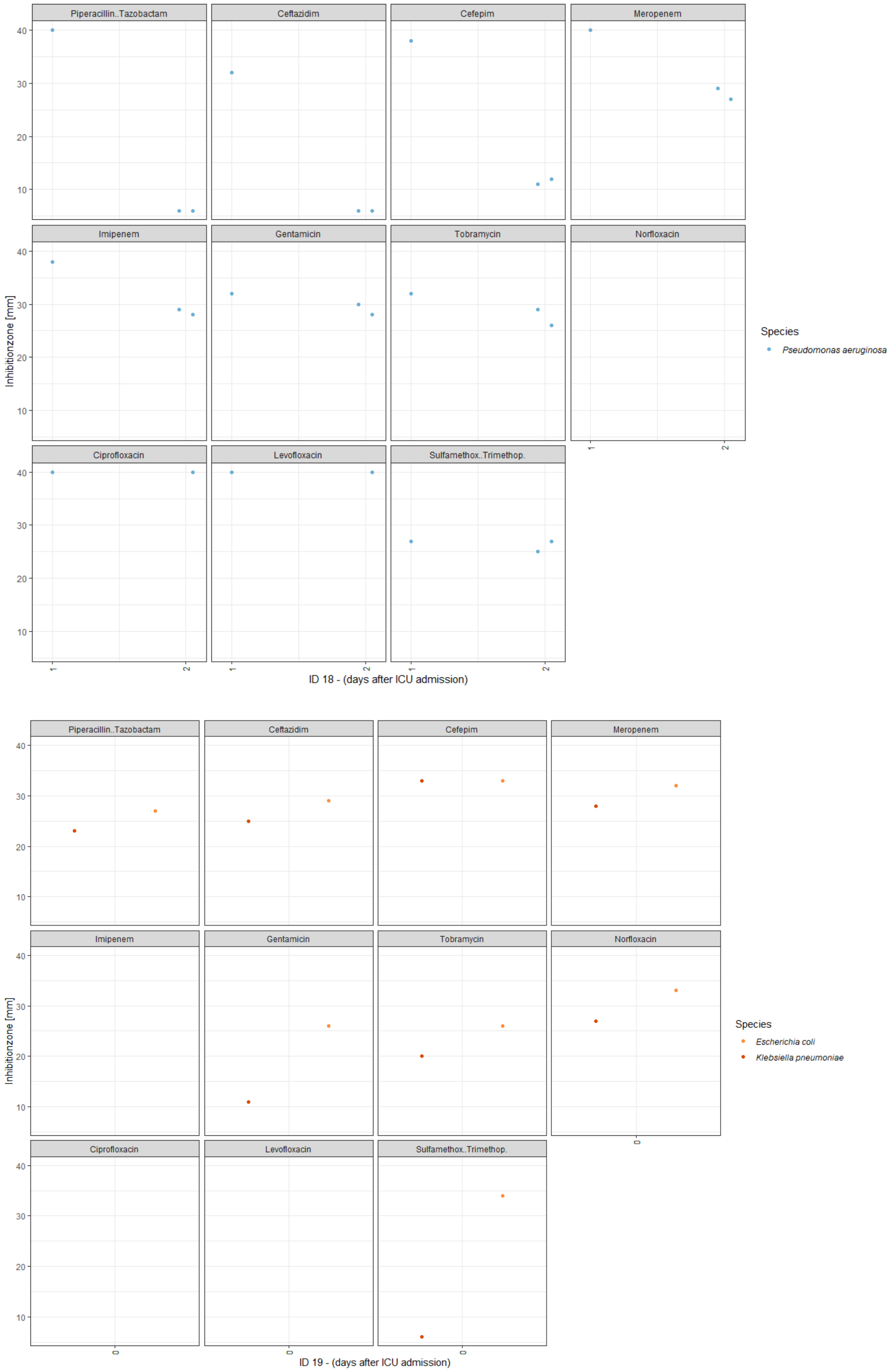

Figure S4. EUCAST disc diffusion inhibition zones of repetitive microbiotaCOVID cohort isolates. ID numbers refer to patient IDs (*n* = 16, only patients with repetitive isolates are shown).

Table S1. Clinical characteristics of patients in this study (*n* = 17).

	***n* = 17**
**Baseline characteristics**	
Age	65 (56-59)
Male sex	14 (82%)
Body mass index (kg/m2)	27.8 (27.2-34.2)
**Clinical data at ICU admission**	
Sepsis-related organ failure assessment score (SOFA)	9 (7-10)
Simplified acute physiology score (SAPS) II	42 (28-51)
**Outcomes**	
Time of pulmonary superinfection detection after ICU admission (days)	7 (3-16)
Duration of ventilation (days)	37 (18-43)
Length of ICU stay (days)	40 (24-51)
Patients died	4 (24%)
**Antimicrobial therapy**	
Empiric antimicrobial therapy at ICU admission	16 (94%)
Beta-lactam/beta-lactamase inhibitors (at any time)	14 (82%)
Cephalosporins (at any time)	15 (88%)
Carbapenems (at any time)	12 (71%)
Aminoglycosides (at any time)	8 (47%)
Quinolones (at any time)	10 (59%)

The data is presented as median (interquartile range (IQR)) or number and percentage (%).

Table S2. Disc diffusion proportion of drug resistant and intermediate isolates.

	Enterobacterales n = 25		*P. aeruginosa n* = 32	
	**Disc diffusion** % resistant (*n*)	**Disc diffusion** % intermediate (*n*)	**Disc diffusion** % resistant (*n*)	**Disc diffusion** % intermediate (*n*)
FEP	8.0% (2)	4.0% (1)	56.3% (18)	43.8% (14) (r./i.)
TZP	32.0% (8)	12.0% (3)	65.6% (21)	34.4% (11) (r./i.)
SXT	4.0% (1)	0.0% (0)	No break point	No break point
MEM	0.0% (0)	0.0% (0)	50.0% (16)	15.6% (5)
AMI	0.0% (0)	(n. ir.)	0.0%	(n. ir.)
TOB	4.0% (1)	(n. ir.)	0.0%	(n. ir.)
GEN	4.0% (1)	(n. ir.)	0.0%	(n. ir.)
PLZ	No DD results	No DD results	No DD results	No DD results
APR	No DD results	No DD results	No DD results	No DD results
AMC	72.0% (18)	0.0% (0)	No DD results	No DD results
CRO	32.0% (8)	0.0% (0)	No DD results	No DD results
CAZ	36.0% (9)	0.0% (0)	46.9% (15)	53.1% (17)
CIP	No DD results	No DD results	15.6% (5)	84.4% (27) (r./i.)

n.ir.: no intermediate disc diffusion range

r./i.: only resistant and intermediate disc diffusion range

cefepime (FEP), piperacillin/tazobactam (TZP), trimethoprim/sulfamethoxazole (SXT), meropenem (MEM), amikacin (AMI), tobramycin (TOB), gentamicin (GEN), plazomicin (PLZ), apramycin (APR), amoxicillin/clavulanate (AMC), ceftriaxone (CRO), ceftazidime (CAZ), ciprofloxacin (CIP)

Table S3. MIC proportion of drug resistant and intermediate isolates.

	Enterobacterales n = 25		*P. aeruginosa n* = 32	
	**MIC** % resistant (*n*)	**MIC** % intermediate (*n*)	**MIC** % resistant (*n*)	**MIC** % intermediate (*n*)
FEP	8.0% (2)	0.0%	65.6% (21)	34.4% (11) (r./i.)
TZP	30.0% (7.5)	6.0% (1.5)	45.3% (14.5)	54.7% (17.5) (r./i.)
SXT	4.0% (1)	0.0% (0)	No break point	No break point
MEM	0.0% (0)	0.0% (0)	50.0% (16)	0.0% (0)
AMI	0.0% (0)	(n. ir.)	0.0% (0)	(n. ir.)
TOB	4.0% (1)	(n. ir.)	0.0% (0)	(n. ir.)
GEN	4.0% (1)	(n. ir.)	0.0% (0)	(n. ir.)
PLZ	0.0% (0)	0.0% (0)	No break point	No break point
APR	0.0% (0)	(n. ir.)	0.0% (0)	(n. ir.)

n.ir.: no intermediate MIC range

r./i.: only resistant and intermediate MIC range

cefepime (FEP), piperacillin/tazobactam (TZP), trimethoprim/sulfamethoxazole (SXT), meropenem (MEM), amikacin (AMI), tobramycin (TOB), gentamicin (GEN), plazomicin (PLZ), apramycin (APR)

Table S4. MIC proportion of drug resistant and intermediate isolates.

	B. cenocepacia n = 12			
	**Disc diffusion** % resistant (*n*)	**Disc diffusion** % intermediate (*n*)	**MIC** % resistant (*n*)	**MIC** % intermediate (*n*)
SXT	0% (0)	0% (0)	0% (0)	(n.ir.)
MEM	0% (0)	0% (0)	8.3% (1)	25% (3)

n.ir.: no intermediate MIC range

trimethoprim/sulfamethoxazole (SXT), meropenem (MEM)
